# TREM2 signaling, miRNA-34a and the extinction of phagocytosis

**DOI:** 10.3389/fncel.2013.00131

**Published:** 2013-08-29

**Authors:** Yuhai Zhao, Walter J. Lukiw

**Affiliations:** Departments of Neurology, Neuroscience, and Ophthalmology, LSU Neuroscience Center, Louisiana State University Health Sciences CenterNew Orleans, LA, USA

**Keywords:** TREM2, miRNA-34a, micro RNA, alzheimer's disease, inflammation, phagocytosis, NF-κB, anti-miRNA

The triggering receptor expressed in myeloid/microglial cells 2 (TREM2; encoded at chr6p21.1) is a glycosylated type 1 transmembrane sensor-receptor of the immunoglobulin-lectin-like gene superfamily expressed in the human central nervous system (CNS). TREM2 normally functions in immune surveillance, sensing and phagocytosis, including the homeostatic clearance of deleterious extracellular debris. Perhaps not too surprising, TREM2 deficiencies have been associated with pathological deficits in phagocytosis, amyloidogenesis and a compromised innate immune system in the inflammatory, neuro-degenerative illnesses polycystic lipomembranous osteodysplasia with sclerosing leukoencephalopathy (PLOSL) and more recently with late onset Alzheimer's disease (AD; Forabosco et al., 2013; Golde et al., 2013; Guerreiro et al., 2013; Jonsson et al., 2013; Neumann and Daly, 2013; Zhao et al., 2013). Meta-analysis from multiple genome-wide association studies (GWAS) in AD have recently identified an rs75932628 (R47H; loss of function) variant in TREM2 as a strong AD risk factor, conveying an increase in AD with an odds ratio of 1.3–8.8-fold (*p* = 0.0076) in recent studies, an effect size comparable to that of the APOEe4 allele (Gonzalez Murcia et al., 2013). However, TREM2 R47H mutations appear to be relatively rare in the human populations so far studied (Gonzalez Murcia et al., 2013; Guerreiro et al., 2013; Hampel and Lista, 2013; Jonsson et al., 2013; Lattante et al., 2013).

**Figure 1:**
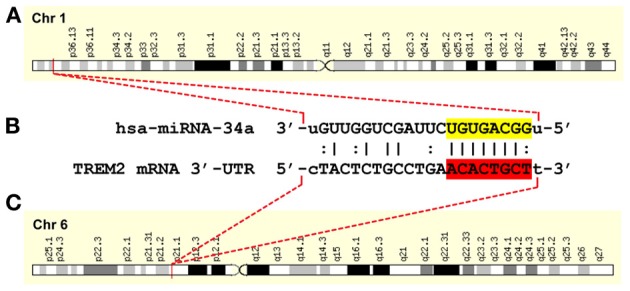
**A hsa-miRNA-34a-TREM2-mRNA-3′-UTR complementarity map; gene products on two independent chromosomes orchestrate a down-regulation of TREM2 and a progressive deficit in cellular debris sensing, phagocytosis and clearance in human neurodegenerative disease. (A)** an NF-κB-sensitive miRNA-34a (encoded at chr1p36.22) and up-regulated in AD has been found to target **(B)** the central domain of the 299 nucleotide human TREM2 mRNA 3′-untranslated region (3′-UTR) of the TREM2 gene **(C)** encoded at chr6p21.1; thus the functional interaction of 2 independent gene products may be responsible for TREM2 deficits in sporadic AD; in**(B)** the miRNA-34a seed sequence 3′-UGUGACGG-5′ is highlighted in yellow; the complementary TREM2-3′-UTR recognition sequence 5′-ACACTGCT-3′ is highlighted in red; an “|” indicates a full hydrogen bond between miRNA-34a and the TREM2-mRNA-30-UTR and a “:” indicates a partial hydrogen bond; the hsa-miRNA-34a recognition feature is located about midway in the TREM2 mRNA-3′-UTR; other miRNA recognition features located within the TREM2-3′-UTR may also affect TREM2 mRNA stability and regulate its expression; other miRNA-mRNA pairings may also be involved in TREM-2 function; the TREM2 gene has no strong NF-κ B binding site within at least 11 kb of its transcription start site (Zhao et al., 2013 and unpublished observations); ribonucleotide sequences and alignment derived using miRBASE algorithms (European Bioinformatics Institute, Wellcome Trust Genome Campus, Hinxton UK; http://www.ebi.ac.uk/enright-srv/microcosm/cgi-bin/targets/v5/detail_view.pl?transcript_id=ENST00000373113; Lukiw, 2013; Neumann and Daly, 2013; Zhao et al., 2013).

Not so rare in AD, however, are significant focal increases in the abundance of a pro-inflammatory, NF-κ B-regulated miRNA-34a (encoded at chr1p36.22) in virtually all AD cells and tissues examined compared to age-matched controls, as well as in amyloid overexpressing transgenic murine models for AD (Schipper et al., 2007; Wang et al., 2009; Zhao et al., 2013). For example, miRNA-34a was recently shown to be up-regulated, and TREM2 was found to be significantly down-regulated, in short post-mortem interval (mean ~2 h) samples of sporadic AD hippocampal CA1 compared with age-matched controls. This novel epigenetic mechanism appears to be mediated by virtue of an unusually strong miRNA-34a recognition feature within the 299 nucleotide TREM2 mRNA 3′-untranslated (3′-UTR) region (energy of association, E_*A*_ ≤ 16 kcal/mol; Figure 1) (Zhao et al., 2013). The stress- and inflammation-induced transcription factor NF-κ B, a driver for miRNA-34a expression, is also strongly up-regulated in the hippocampal CA1, and both NF-κ B inhibitors and stabilized anti-miRNA-34a are effective in restoring TREM2 back to homeostatic levels (Kaltschmidt and Kaltschmidt, 2009; Lukiw, 2013; Zhao et al., 2013). Interestingly, a pathologically up-regulated miRNA-34a has been strongly associated with progressive neurotrophic deficits (Wang et al., 2009), altered synaptogenesis (Agostini et al., 2011) and deficient immune and phagocytotic responses in inflammatory degenerative disorders such as cardiovascular disease (Boon et al., 2013), multiple sclerosis (Junker et al., 2009), and in sporadic AD mononuclear cells (Schipper et al., 2007) as well as in AD brain (Zhao et al., 2013).

Abundant evidence indicates that multiple genes, through multiple genetic processes, initiate and propagate AD-type change. Collectively, emerging observations indicate that an epigenetic mechanism involving an NF-κB-mediated, miRNA-34a-regulated down-regulation of TREM2 expression may shape innate immunity, inflammation and the extinction of the phagocytic response that contributes to amyloidogenesis and inflammatory neurodegeneration. Pro-inflammatory transcription factors and miRNAs, such as NF-κ B and miRNA-34a, and their target mRNA 3′-UTRs appear to form a highly interwoven genetic regulatory network that may escape classical GWAS- and SNP-based detection. Interestingly, AD-relevant stress-mediated up-regulation of miRNA-34a in cultured microglial cells, subsequent down-regulation in the expression of TREM2-3′-UTR reporter vectors, and rescue by stabilized anti-miRNA-34a indicates that this type of pathogenic signaling can be effectively quenched, at least *in vitro* (Lukiw, 2013; Zhao et al., 2013). Totally novel anti-miRNA strategies involving miRNA-34a mimics (i.e., MRX34) that normally induce senescence and apoptosis, and utilizing liposome delivery technologies are just now appearing in the clinic for the treatment of metastatic liver cancer (Bouchie, 2013). In the near future these approaches may have considerable potential in also directing novel, combinatorial anti-NF-κ B- and/or anti-miRNA-based AD therapeutic strategies that target the multiple pathogenic pathways which lie at the core of the AD process.
